# Sulforaphene inhibits esophageal cancer progression via suppressing SCD and CDH3 expression, and activating the GADD45B-MAP2K3-p38-p53 feedback loop

**DOI:** 10.1038/s41419-020-02859-2

**Published:** 2020-09-01

**Authors:** Sichong Han, Yandong Wang, Jie Ma, Zhe Wang, Hui-Min David Wang, Qipeng Yuan

**Affiliations:** 1 State Key Laboratory of Chemical Resource Engineering, Beijing University of Chemical Technology, Beijing, 100029 P.R. China; 2 Department of Biotherapy, Beijing Hospital, National Center of Gerontology, Chinese Academy of Medical Sciences & Peking Union Medical College, Beijing, 100730 P.R. China; 3 Graduate Institute of Biomedical Engineering, National Chung Hsing University, Taichung City, 402 Taiwan; 4 Department of Medical Laboratory Science and Biotechnology, China Medical University, Taichung City, 404 Taiwan; 5 Graduate Institute of Medicine, College of Medicine, Kaohsiung Medical University, Kaohsiung City, 807 Taiwan; 6 College of Food and Biological Engineering, Jimei University, Xiamen City, 361021 Fujian Province P.R. China; 7 Undergraduate Program Study of Biomedical Engineering, Physics Department, Airlangga University, Surabaya City, 60115 Indonesia

**Keywords:** Oesophageal cancer, Oncogenes, Tumour-suppressor proteins

## Abstract

Esophageal cancer is one of the most common cancer with limited therapeutic strategies, thus it is important to develop more effective strategies to against it. Sulforaphene (SFE), an isothiocyanate isolated from radish seeds, was proved to inhibit esophageal cancer progression in the current study. Flow cytometric analysis showed SFE induced cell apoptosis and cycle arrest in G2/M phase. Also, scrape motility and transwell assays presented SFE reduced esophageal cancer cell metastasis. Microarray results showed the influence of SFE on esophageal cancer cells was related with stearoyl-CoA desaturase (SCD), cadherin 3 (CDH3), mitogen-activated protein kinase kinase 3 (MAP2K3) and growth arrest and DNA damage inducible beta (GADD45B). SCD and CDH3 could promote esophageal cancer metastasis via activating the Wnt pathway, while the latter one was involved in a positive feedback loop, GADD45B-MAP2K3-p38-p53, to suppress esophageal cancer growth. GADD45B was known to be the target gene of p53, and we proved in this study, it could increase the phosphorylation level of MAP2K3 in esophageal cancer cells, activating p38 and p53 in turn. SFE treatment elevated MAP2K3 and GADD45B expression and further stimulated this feedback loop to better exert antitumor effect. In summary, these results demonstrated that SFE had the potential for developing as a chemotherapeutic agent because of its inhibitory effects on esophageal cancer metastasis and proliferation.

## Introduction

Esophageal cancer is the top 10 global arising common cancer and multiple studies suggest that excessive smoking, hot tea and red meat consumption, poor oral health, and low intake of fresh fruits and vegetables are associated with a high risk of it. Because of esophageal cancer mostly diagnosed in advanced late stages, the general prognosis is unsatisfied with an average of more than 200,000 deaths per year happening^1^. As current treatment strategies are limited, it remains urgent to develop more efficient therapies to control it.

With the development of research on cancer prevention and treatment, accumulated epidemiological evidence indicated that many natural active substances, such as astaxanthin^2^ and resveratrol^3^ had inhibitory effects on multiple cancers. Among them, isothiocyanates from cruciferous vegetables have attracted more and more attention. Sulforaphene (SFE), as an isothiocyanate isolated from radish seeds^4,5^, was proved to possess strong anticarcinogenic activities. It could induce apoptosis in lung cancer cell lines by inhibiting the PI3K-Akt pathway^6^ and cause hepatocellular carcinoma cell death through repressing keratin 8 and activating anoikis^7^. However, it is still not clear whether SFE also function as an antineoplastic compound in esophageal cancer.

Stearoyl-CoA desaturase (SCD) has been shown to promote lung cancer growth by increasing monounsaturated fatty acids level^8^ and to activate the endoplasmic reticulum unfolded protein response^9^, which was associated with cancer cell metastasis^10^. Cadherin 3 (CDH3), a classical cadherin of the cadherin superfamily, has also been linked to many types of cancer, such as colorectal, breast, and pancreatic cancers^11–13^. Mitogen-activated protein kinase kinase 3 (MAP2K3) belongs to the MAP kinase kinase family and participates in the MAP kinase-mediated signaling cascade. It activates p38 to play its role in hepatocellular carcinoma^14^, colorectal cancer^15^, breast cancer^16^ and other tumors. Growth arrest and DNA damage inducible beta (GADD45B), a member of the GADD45 gene family which is induced by various genotoxic stresses^17–19^, is involved in DNA damage repair, cell cycle arrest, and cell survival. It has a strong inhibitory effect on proliferation and metastasis in multiple tumors such as hepatocellular carcinoma^20^. According to microarray results, we confirmed these four genes were the targets of SFE in esophageal cancer cells, and SFE could inhibit esophageal cancer progression through suppressing SCD and CDH3 expression, and activating the GADD45B-MAP2K3-p38-p53 feedback loop.

In conclusion, our findings identify the key genes and signaling pathways involved in SFE inhibiting metastasis and proliferation of esophageal cancer. These results reveal the mechanism of SFE against esophageal cancer, and suggest that SFE has great potential to be applied as an anticancer agent.

## Results

### SFE inhibits esophageal cancer cell proliferation and metastasis

**Fig. 1:**
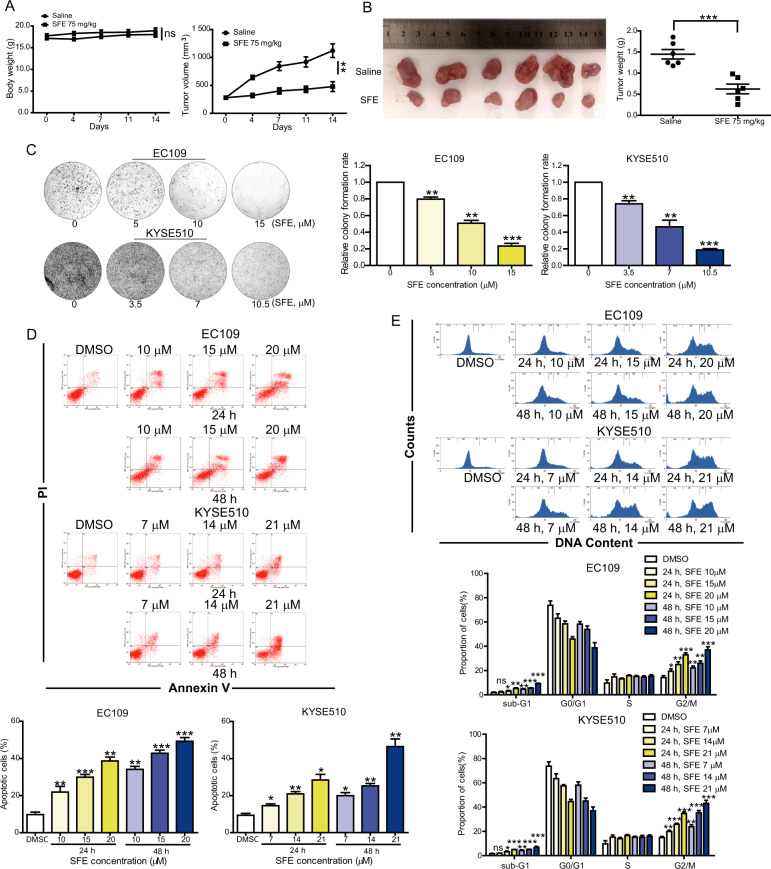
SFE inhibits esophageal cancer proliferation in vivo and in vitro. **a** Mice body weight and tumor volume were measured every 3 days after injection of SFE or saline. **b** Image of tumor lumps removed from nude mice (*n* = 6) injected saline or SFE (75 mg/kg) and the weight of harvestes transplanted tumors. **c** Colony formation assay was carried out in SFE-treated EC109 and KYSE510 cells. **d**, **e** EC109 and KYSE510 cells were treated with gradient concentration of SFE for 24 h and 48 h, respectively, followed by using flow cytometry to asses the apoptotic rates (**d**) and cell cycle distribution (**e**). Data represent the mean ± s.d. of three independent experiments. The statistical significance was assessed by Student’s *t*-test. **P < 0.01 and ***P < 0.005. ns, not significant.

**Fig. 2:**
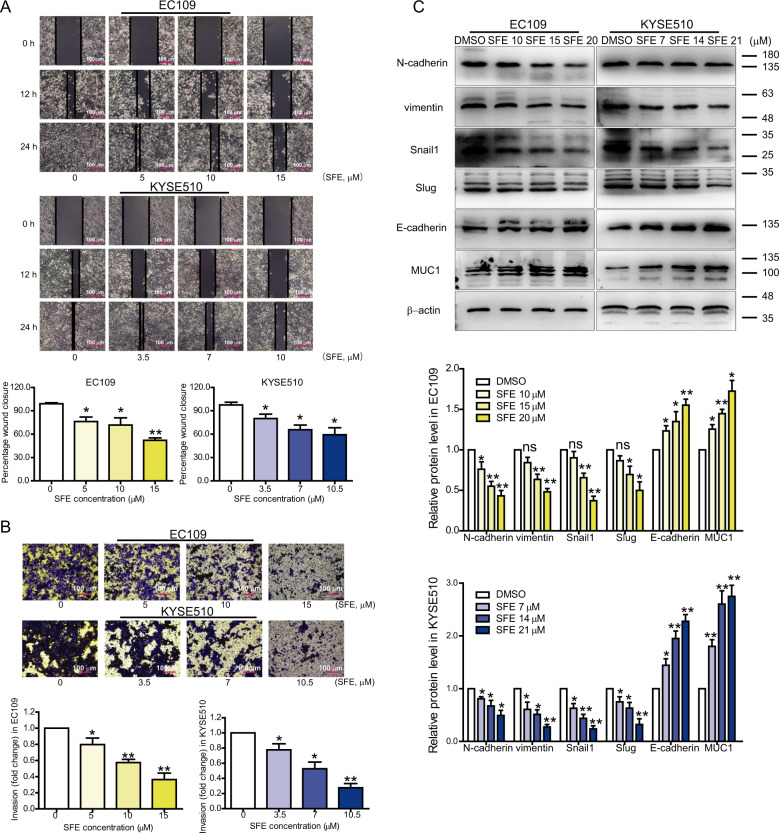
SFE suppresses esophageal cancer cell metastasis. **a**, **b** Scrape motility assay (**a**) and transwell assay (**b**) were performed in SFE-treated EC109 and KYSE510 cells. **c** The RNA and protein levels of EMT markers in SFE-treated EC109 and KYSE510 cells were analyzed by qRT-PCR (upper panel) and western blotting (lower panel). Original magnification ×100. Scale bars = 100 μm. Data represent the mean ± s.d. of three independent experiments. The statistical significance was assessed by Student’s *t*-test. *P < 0.05 and **P < 0.01. ns, not significant.

To evaluate the effect of SFE on esophageal cancer in vivo, KYSE150 cells xenograft model was established. There was no significant difference in mice body weight, while the tumor volume and lumps weight in mice treated with SFE were obviously smaller than that of the control group (Fig. 1a, b). We then examined the role of SFE in four esophageal cancer cell lines, finding a dose-dependent inhibition of SFE on cell proliferation (Supplementary Fig. S1A). EC109 and KYSE510 cells were more sensitive to SFE and selected for the following experiments. The anti-proliferative activity of SFE was further determined using the colony formation assay, which demonstrated a decreasing trend of relative colony formation rates of cancer cells as the SFE concentration increased gradually (Fig. 1c). We next examined whether this suppressed effect was due to cell apoptosis and cell cycle arrest. Fluorescence-activated cell sorting (FACS) analysis showed a significantly higher level of apoptosis in SFE-treated EC109 and KYSE510 cells than that in DMSO-treated cells (Fig. 1d). Caspases are proteolytic enzymes widely known for modulating cell death, and we measured the activity of the most critical ones, finding caspase-9 and caspase-3 activities were significantly elevated, yet that of caspase-8 was almost unchanged (Supplementary Fig. S1B). As the mitochondrial pathway and the death receptor pathway are reported to mediate caspases activation, and caspase-9 and caspase-8 belonged to the two pathways respectively^21,22^, we predicted SFE induced mitochondrial apoptosis in esophageal cancer cells and then measured the mitochondrial membrane potential, the decrease of which was a characteristic performance in mitochondrial apoptosis. The result shown in Supplementary Fig. S1C was consistent with our prediction. Western blotting detection of mitochondrial apoptosis-related protein levels further proved this prediction (Supplementary Fig. S1D). Apart from cell apoptosis, SFE treatment could induced G2/M cell cycle arrest in a dose-dependent and time-dependent manner (Fig. 2e and Supplementary Fig. S1E). The change of sub-G0 phase shown in Fig. 2e also indirectly indicated SFE induced esophageal cancer cell apoptosis dose-dependently and time-dependently.

SFE treatment also suppressed esophageal cancer cell metastasis (Fig. 2a, b). It is widely accepted that epithelial-mesenchymal transition (EMT) has a major impact on cancer metastasis, for the weak cell-cell interaction and high migratory ability caused by EMT assisting cells to isolate from primary lesions to blood vessels. So we detected EMT markers expression, and found epithelial cell-related protein levels were promoted by SFE treatment while those of mesenchymal cell-related protein were significantly downregulated (Fig. 2c).

### SFE inhibits cell metastasis through inactivation of the Wnt pathway

**Fig. 3:**
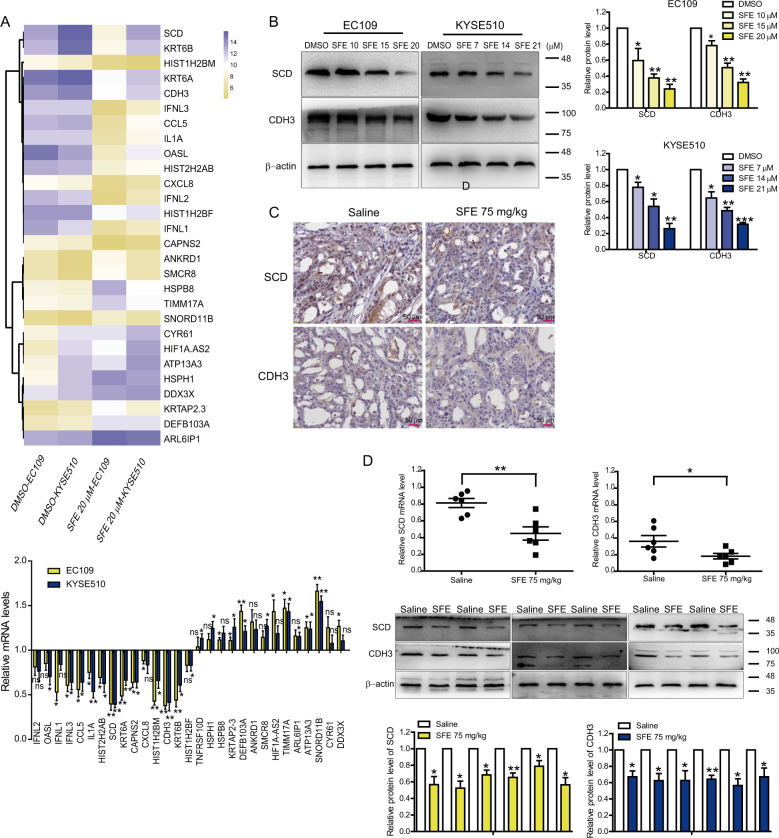
SCD and CDH3 are the target genes of SFE in esophageal cancer cells. **a** (upper) The top 30 upregulated and downregulated mRNAs in both EC109 and KYSE510 cell lines were selected for further experiments. (lower) qRT-PCR in EC109 and KYSE510 cells treated with 20 µM SFE or DMSO. **b** Western blotting showed the effect of SFE on SCD and CDH3 protein abundance in EC109 and KYSE510 cells. **c** Representative images of immunohistochemistry which demonstrated SCD and CDH3 protein levels in saline- or SFE-treated xenograft tumor lumps. **d** The relative expression of SCD and CDH3 in vivo was analyzed by qRT-PCR and western blotting, respectively. Original magnification ×400. Scale bars = 60 μm. Data represent the mean ± s.d. of three independent experiments. The statistical significance was assessed by Student’s *t*-test. *P < 0.05, **P < 0.01, and ***P < 0.005. ns, not significant.

To identify the target genes and pathways related to SFE regulating esophageal cancer peogression, EC109 and KYSE510 cells treated with SFE or DMSO were analyzed via microarrays. We selected the coincident mRNAs between the top 30 upregulated and downregulated ones in the two cell lines for further validation (Fig. 3a, upper), and found that SCD and CDH3 mRNA levels decreased most significantly in SFE-treated cells (Fig. 3a, lower). Proving SFE could decrease SCD and CDH3 expression in vitro and in vivo (Fig. 3b–d), we identified SCD and CDH3 were the targets of SFE in esophageal cancer cells.

**Fig. 4:**
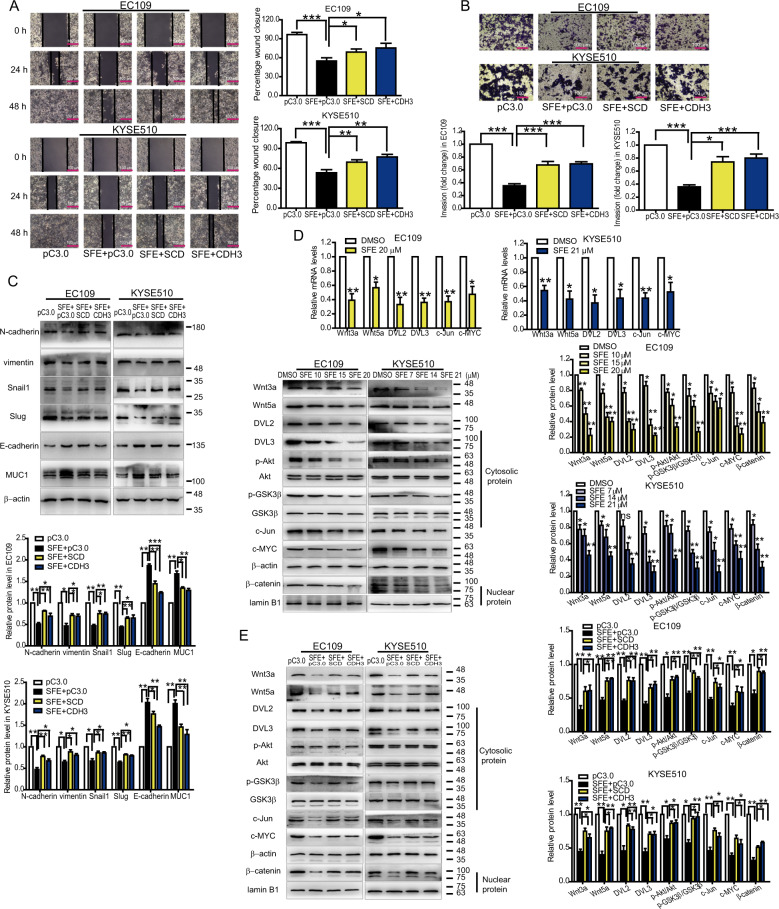
SFE inactivates the Wnt pathway by downregulating SCD and CDH3 expression. **a**, **b** Scratch motility assay (**a**) and transwell assay (**b**) in EC109 and KYSE510 cells. **c** Western blotting detection of EMT related gene expression in EC109 and KYSE510 cells. **d** qRT-PCR and western blotting were used to show the mRNA and protein levels of the Wnt pathway-related genes in SFE-treated EC109 and KYSE510 cells. **e** Western blotting detection of the Wnt pathway-related gene expression after overexpressing SCD and CDH3 in SFE-treated EC109 and KYSE510 cells. Original magnification ×100. Scale bars = 100 μm. Data represent the mean ± s.d. of three independent experiments. The statistical significance was assessed by Student’s *t*-test. *P < 0.05, **P < 0.01, and ***P < 0.005. ns, not significant.

Next, we tested whether SCD and CDH3 played an important role in esophageal cancer cells. siRNAs specific for SCD and CDH3, as well as plasmids harboring full-length human SCD and CDH3 sequences, were designed and transfected into cancer cells (Supplementary Fig. S2A, B). It was found that both SCD and CDH3 had no influence on cell proliferation (Supplementary Fig. S2C, D) but could promote cell metastasis (Supplementary Fig. S3A, B). Moreover, overexpressing SCD and CDH3 could reverse the inhibition of SFE on cell metastatic capacity (Fig. 4a–c), indicating SCD and CDH3 were involved in SFE suppressing cell metastasis.

To reveal the mechanism by which SCD and CDH3 increased the metastatic ability of esophageal cancer cells, we searched on the LinkedOmics database (http://www.linkedomics.org/) and found both SCD and CDH3 expression were positively correlated with the Wnt pathway widely known to be associated with cell metastasis (Supplementary Fig. S4A, B)^23–25^, which was verified by qRT-PCR and western blotting (Supplementary Fig. S5A, B). Then we verified SFE could inactivate the Wnt pathway (Fig. 4d) while SCD and CDH3 overexpression reactivated it in esophageal cancer cells (Fig. 4e). In short, SFE inactivated the Wnt pathway by downregulating SCD and CDH3 expression to suppress esophageal cancer cell metastasis.

### SFE inhibits cell proliferation through a positive feedback loop

**Fig. 5:**
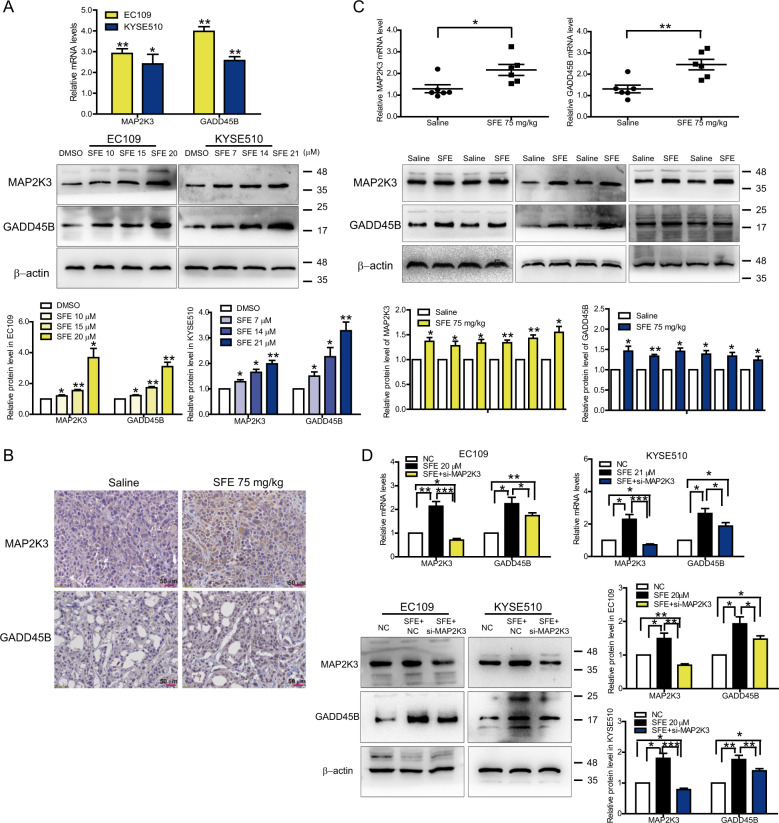
MAP2K3 and GADD45B are the target genes of SFE in esophageal cancer cells. **a** The relative expression of MAP2K3 and GADD45B in SFE-treated EC109 and KYSE510 cells was analyzed by qRT-PCR and western blotting, respectively. **b** Representative images of immunohistochemistry showed the MAP2K3 and GADD45B protein levels in saline-treated or SFE-treated xenograft tumor lumps. **c** The relative expression of MAP2K3 and GADD45B in vivo was analyzed by qRT-PCR and western blotting, respectively. **d** qRT-PCR and western blotting in EC109 and KYSE510 cells. Original magnification ×400. Scale bars = 60 μm. *si-MAP2K3*, equal mixed si-MAP2K3-1 and si-MAP2K3-2. NC: negative control RNA duplex, the control of siRNA. Data represent the mean ± s.d. of three independent experiments. The statistical significance was assessed by Student’s *t*-test. *P < 0.05 and **P < 0.01.

We had proven that SFE could significantly inhibit esophageal cancer cell progression, and SCD and CDH3 were involved in SFE inhibition of cell metastasis, yet the mechanism by which SFE inhibited cell proliferation was still unclear. So we compared the KEGG enrichment analysis of microarray data and found the p53 pathway was one of the top 30 pathways in both analyses (Supplementary Fig. S6A). Interestingly, SFE improved p53 protein level rather than transcriptionally regulated p53 gene (Supplementary Fig. S6B), suggesting that there were other effectors assisting SFE to regulate the p53 pathway. p38 had been proved to phosphorylate p53 on Ser33 and Ser46 as a prominent activator^26–28^, and we found MAP2K3 and GADD45B, the activators of p38^14–16,29,30^, were differentially expressed in the microarray of EC109 cells. After confirming SFE could upregulate MAP2K3 and GADD45B expression in vitro and in vivo (Fig. 5a–c), and considering GADD45B was the target gene of p53^31–33^, we guessed there was a specific cascade, MAP2K3/GADD45B-p38-p53-GADD45B, in SFE-treated esophageal cancer cells. Western blotting certified this axis made sense (Supplementary Fig. S6C). After identifying 30 µM was the optimal concertration of SB202190 (Supplementary Fig. S6D), a commonly used inhibitor of the p38 pathway^34–36^, we applied SB202190 and SFE successively to process esophageal cancer cells and further verified p38, p53 and GADD45B could be reactivated by SFE (Supplementary Fig. S6E). Yet these results implied GADD45B expression was not necessarily directly regulated by SFE. siRNAs specific for MAP2K3 were designed (Supplementary Fig. S7A, B) and transfected into SFE-treated cancer cells to determine the relationship between SFE and GADD45B. The results demonstrated that MAP2K3 expression was lower than that of the negative control group while GADD45B expression was still higher (Fig. 5d), proving GADD45B was indeed the target of SFE. Through transfection of siRNAs specific for MAP2K3 and GADD45B, as well as plasmids harboring full-length human MAP2K3 and GADD45B sequences (Supplementary Fig. S7A, B), we found MAP2K3 and GADD45B inhibited esophageal cancer cell proliferation (Supplementary Fig. S7C, D). Furthermore, decreasing MAP2K3 and GADD45B expression could rescue the cell apoptosis and G2/M arrest induced by SFE (Supplementary Fig. S8A–D), indicating MAP2K3 and GADD45B were related to SFE inhibiting cell proliferation.

**Fig. 6:**
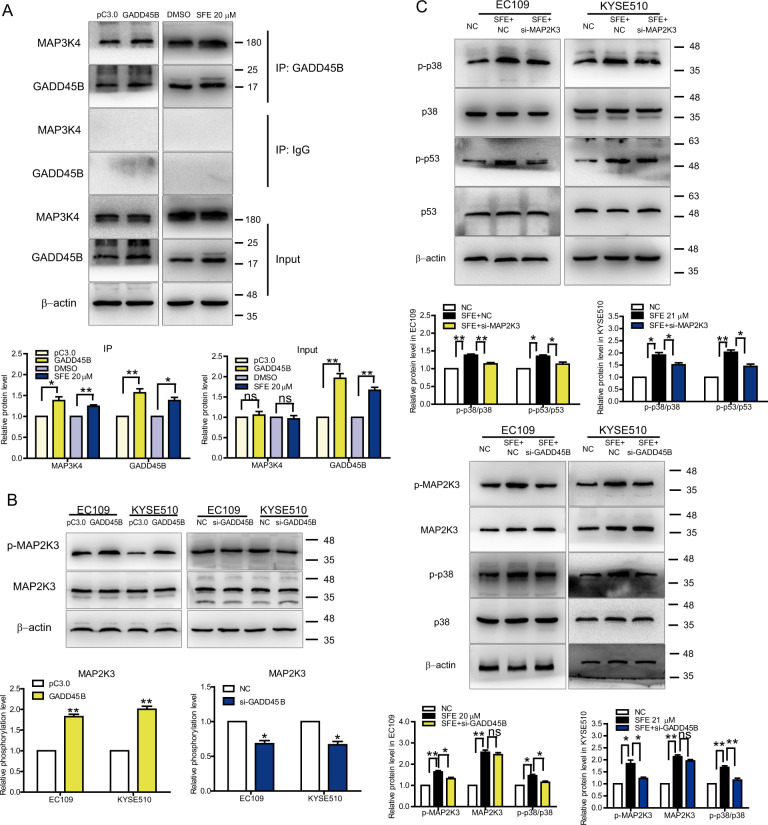
A GADD45B-MAP2K3-p38-p53 positive feedback loop in SFE-treated esophageal cancer cells. **a** co-IP assay was carried out in EC109 cells with GADD45B overexpressing or SFE treatment (20 μM). **b** The phosphorylation level of MAP2K3 in EC109 and KYSE510 cells with GADD45B overexpression or expression decreased was detected by western blotting. **c** After decreasing MAP2K3 and GADD45B expression in SFE-treated EC109 and KYSE510 cells, the p38 pathway-related protein levels were detected by western blotting. *si-MAP2K3*, equal mixed si-MAP2K3-1 and si-MAP2K3-2. *si-GADD45B*, equal mixed si-GADD45B-1 and si-GADD45B-2. NC: negative control RNA duplex, the control of siRNA. Data represent the mean ± s.d. of three independent experiments. The statistical significance was assessed by Student’s *t*-test. *P < 0.05 and **P < 0.01. ns, not significant.

It was reported that GADD45B could bind and relieve the autoinhibition of MAP3K4^37–39^, an activator of MAP2K3^40^, and the STRING database (http://string-db.org/) also pointed out the interaction among GADD45B, MAP3K4, and MAP2K3 (Supplementary Fig. S9, left). Thus we speculated that there might be a positive feedback loop composed of GADD45B, MAP2K3, p38, and p53 (Supplementary Fig. S9, right). To confirm this speculation, We carried out co-IP assay and found more MAP3K4 was immunoprecipitated when GADD45B was overexpressed in EC109 cells (Fig. 6a). As we had proved that GADD45B was a target of SFE, SFE treatment could induce the same result shown in Fig. 6a. Based on the conclusion that MAP3K4 could be bound to GADD45B (Fig. 6a) and activated MAP2K3 subsequently (Fig. 6b), and SFE activation of the p38 pathway was partially abrogated by decreasing MAP2K3 and GADD45B expression (Fig. 6c), we could summarize that the inhibitory effect of SFE on cell proliferation was achieved by the GADD45B-MAP2K3-p38-p53 positive feedback loop.

**Fig. 7:**
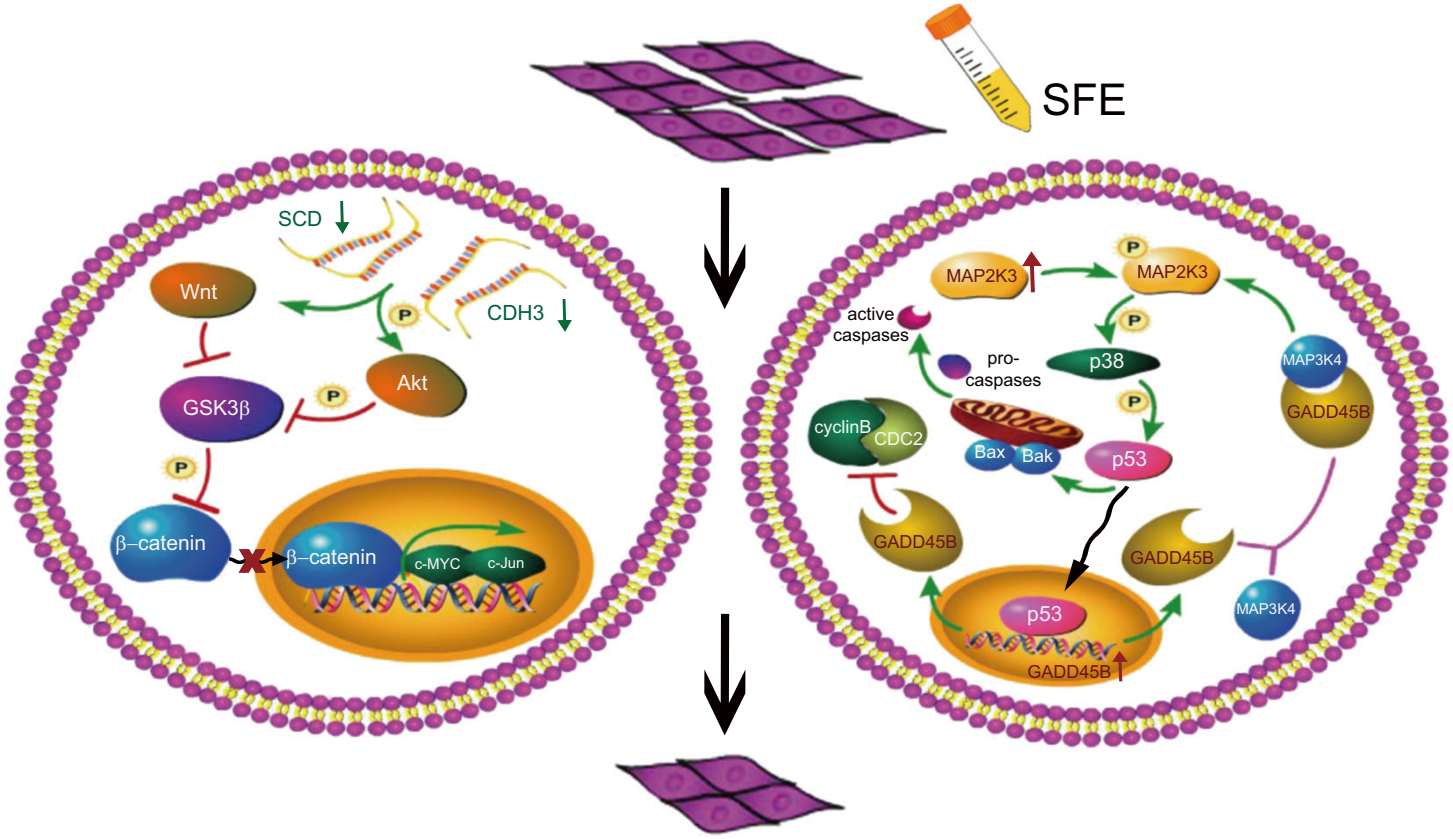
Schematic representation illustrates SFE modulation of esophageal cancer cell metastasis and proliferation. It illustrates the mechanism by which SFE inhibits esophageal cancer metastasis and proliferation.

A schematic representation of the association between SFE and esophageal cancer cells was illustrated in Fig. 7. It described that SFE inactivated the Wnt pathway through suppression of SCD and CDH3 expression to inhibit cell metastasis, and by activating the GADD45B-MAP2K3-p38-p53 positive feedback loop, SFE had an inhibitory influence on cell proliferation.

## Discussion

Esophageal cancer is one of the most common cancer with extremely aggressiveness and poor survival rate. Patients underwent therapeutic surgeries have a 5-year survival rate of only 25%, even worse when they are not suitable for therapeutic surgeries. More and more evidence suggest that isothiocyanates from cruciferous vegetables is associated with a decreased risk of various cancers^2–4^. As one of the isothiocyanates, SFE has attracted much attention for its potential role in protecting people from multiple diseases. This study revealed the strong antitumor activity of SFE and its mechanism of inhibiting esophageal cancer progression.

So far lots of reports have mentioned that the Wnt pathway is associated with cell metastasis, repression of which could prevent EMT and then inhibit metastasis^23–25^. In this study, we showed that SFE could inactivate it through inhibiting SCD and CDH3 expression to regulate esophageal cancer cell metastasis. However, we are unable to figure out the effectors which interact with SCD and CDH3 directly to achieve their regulation of the Wnt pathway, thus further studies are warranted to deeply investigate the regulatory mechanism.

In addition, we found SFE could induce G2/M arrest and cell apoptosis by up-regulating MAP2K3 and GADD45B expression to activate p38 and p53 in order. The role of the p38 pathway in cancer development seems to be uncertain. Some studies demonstrated that p38 mediated cancer cell metastasis to promote tumorigenesis^41^ while some showed that the p38 pathway acted as a tumor suppressor^42^. In our study, p38 played a negative role in esophageal cancer cell proliferation. It was reported that MAP3K4 could be bound to and activated by GADD45B through disrupting its N-terminal noncatalytic domain inhibition of the C-terminal kinase domain^37–39^. We used co-IP assay to confirm this interaction for the first time in esophageal cancer cells and identified the GADD45B-MAP2K3-p38-p53 cascade was highly associated with the inhibition of SFE on cell proliferation. Studies had shown that apoptosis-related proteins were involved in regulating cancer cell metastasis, for example, pro-survival Bcl-2 over-expression was associated with enhanced cell invasion and migration^43,44^ while pro-apoptotic Bad and Bax could significantly suppress EMT^45^. As SFE induced mitochondrial apoptosis through the activated p53, and several observations implied p53 could inactivate the Wnt pathway in various ways^46–48^, and we speculated that there was a connection between cell apoptosis caused SFE-treatment and metastasis, which involved p53 and the Wnt pathway in esophageal cancer cells. In the coming future, we will verify our guess that SFE-mediated cell metastasis may be attributed to cell apoptosis.

In summary, our study emphasizes that SFE inhibits esophageal cancer progression via suppressing SCD and CDH3 expression, and activating the GADD45B-MAP2K3-p38-p53 positive feedback loop. To the best of our knowledge, this is the first study to reveal the anti-esophageal cancer mechanism of SFE and characterize the specific roles of SCD, CDH3, MAP2K3, and GADD45B in esophageal cancer cells. These results provide new insights into the strong antineoplastic activity of SFE and prove SFE a potential chemotherapeutic agent to overcome esophageal cancer.

## Materials and methods

### Cell culture and chemicals

The human esophageal cancer EC109, KYSE510, KYSE150, and TE-1 cell lines were obtained from the National Infrastructure of Cell Line Resource, cultured in RPMI-1640 medium (Gibco, Grand Island, NY, USA) with 10% fetal bovine serum (FBS) (Gibco), 100 units/mL penicillin (Invitrogen, Carlsbad, CA, USA) and 100 mg/mL streptomycin (Invitrogen). The cell lines were characterized by Genetic Testing Biotechnology Corporation (Suzhou, China) using short tandem repeat markers and they were not contaminated by mycoplasma detected by Myco-Lumi Luminescent Mycoplasma Detection Kit (Beyotime, Shanghai, China). SFE was separated and purified from radish seeds (Beijing Tongrentang Co., LTD, Beijing, China) as reported previously^2,3^, dissolved in DMSO (Beijing Chemical Factory, Beijing, China). SB202190 was obtained from MedChem Express (Monmouth Junction, NJ, USA).

### Xenograft tumor assay

KYSE150 xenograft in 6-week-old female nude BALB/c mice (Beijing Weitong Lihua Experimental Animal Technology Co., Ltd) was established. 8.0×10^6^ KYSE150 cells suspended in RPMI-1640 medium were subcutaneously inoculated into fossa axillaris of mice. When the tumor volume reached 100–300 mm^3^, mice with too large or too small tumors were eliminated. Eventually, 12 mice bearing similar volume of tumors were selected and randomly divided into two groups and treated with 75 mg/kg SFE or saline via intraperitoneal injection once a day for 2 weeks. Body weight and tumor volume were measured twice a week after the first injection. At the end of treatment, all mice were sacrificed, and tumors were imaged, weighed, and dissected prior to immunohistochemical analysis (Supplementary Table S1). The operators and investigators were blinded to the group allocation throughout the process. Immunoreactivity was detected by a horseradish peroxidase kit (BioGenex, Fremont, CA, USA). Then the slides were counterstained with hematoxylin, dehydrated and fixed.

This assay was carried out in accordance with an Institutional Animal Care and Use Committee (IACUC) of Institute of Biophysics, Chinese Academy of Sciences and performed in accordance with the Guidelines for Animal Experiments of IACUC according to the laws and notifications of the People’s Republic of China.

### Plasmid and small RNAs construction

Full-length homo sapiens SCD, CDH3, MAP2K3, and GADD45B were cloned into the pcDNA3.0 (pC3.0) plasmid, producing pC3.0-SCD, pC3.0-CDH3, pC3.0-MAP2K3, and pC3.0-GADD45B plasmids. All of the plasmids, as well as small interfering RNA (siRNA) duplexes and negative control RNA duplex (NC) (Supplementary Table S2), were purchased from Genepharma.

### Cell viability assay

Cell viability was measured using the Cell Counting Kit-8 (CCK-8) reagent (Beyotime) according to the manufacturer’s instructions. Cells seeded in 96-well plates were treated with DMSO or gradient concentrations of SFE for 48 h. After CCK-8 solution was added to each well, cells were incubated at 37 °C for 1 h. The absorbance was measured by microplate reader (Bio-Rad, Hercules, CA, USA) at 450 nm.

### Colony formation assay

Cells were treated with gradient concentrations of SFE, or transfected with plasmids or siRNAs specific for SCD, CDH3, MAP2K3 and GADD45B. After 9 days, cells were fixed with 3.7% formaldehyde and dyed with crystal violet. The colony numbers were counted and analyzed by ImageJ 2X software (Rawak Software, Inc. Germany). DMSO treatment, pC3.0 plasmid and NC were used as negative control, respectively.

### Cell counting, apoptosis, and cell cycle analysis

For cell counting, cells were transfected with plasmids or siRNAs. After 24 h, 48 h, and 72 h, they were harvested and counted, respectively. Cells treated with SFE or transfected with siRNAs in combination with SFE treatment, were collected after 24 h and 48 h, followed by cell apoptosis and cell cycle analysis. Cell apoptosis was determined using the Dead Cell Apoptosis Kit with Annexin V Alexa Fluor™ 488 & Propidium Iodide (Invitrogen) and cell cycle distribution was analyzed with Cell Cycle Detection Kit (KeyGEN BioTECH, Jiangsu, China) according to the manufacturer’s instructions. Both analyses were detected with MoFlo XDP flow cytometer (Beckman Coulter, Miami, FL, USA) and data was processed by Summit V5.2.1 (Beckman Coulter).

### caspase-8/caspase-9/caspase-3 activity assay

Caspase activity was measured with Caspase-8 Activity Assay Kit, Caspase-9 Activity Assay Kit, and Caspase-3 Activity Assay Kit (Beyotime) according to the manufacturer’s instruction. Cells were lysed after exposed to gradient concentrations of SFE for 24 h and 48 h, respectively. Substrates were diluted to 0, 10, 20, 50, 100, and 200 µM as standards. Cell lysates were mixed with 10 µL of 2 mM substrate and reaction buffer to a total volume of 100 µL, and incubated at 37 °C for 2 h. Absorbance of samples and standards was measured with microplate reader (Bio-Rad) at 405 nm.

### Measurement of mitochondrial membrane potential

This assay was accomplished with Mitochondrial Membrane Potential Assay Kit with JC-1 (Beyotime). In brief, cells were treated with gradient concentrations of SFE for 24 h, or the same concentration of SFE for 6 h, 12 h and 24 h, respectively. 10 µM CCCP was added to a well as a positive control and the plates were incubated for 20 min. Then cells were treated with JC-1 staining solution and incubated for another 20 min at 37 °C. Finally, the plates were sealed and observed on a fluorescence microscope (80i, Nikon). Images were analyzed by ImageJ 2X software.

### Scrape motility and transwell assays

In scrape motility assay, cells were scratched with a sterile 100 µl pipette tip and photographed at ×100 magnification using BEION medical image software V4.20 (Beion, Shanghai, China) at different time points. In transwell assay, the transwell chambers (Corning, NY, USA) were covered with matrigel (BD Biosciences, San Jose, CA, USA) overnight. Cells cultured in 1% FBS were added to the chambers and medium with 10% FBS was added to the lower wells. After 48 h incubation, the number of cells invading through the matrigel was counted in 6 randomly selected visual fields using a Leica DM3000 microscope (Leica, Wetzlar, Germany). Data were analyzed by ImageJ 2X software.

### Microarray analysis

Total RNA was extracted from EC109 and KYSE510 cells treated with SFE (20 µM) or DMSO as negative control. Analyses were achieved with Affymetrix Human Transcriptome Array 2.0 by Shanghai Biotechnology Corporation. Fold change > 2 and *P*-value < 0.05 were set as the threshold for significantly differential expression. The heat map drawn by ImageGP (www.ehbio.com/ImageGP/index.php/Home/Index/index.html) showed the top 30 upregulated and downregulated mRNAs in both cell lines which were selected for further research. Related signaling pathways were selected based on Kyoto Encyclopedia of Genes and Genomes (KEGG) enrichment analysis. The microarray data are deposited in the NCBI Gene Expression Omnibus (GEO) datasets under the accession number GSE150891.

### Quantitative reverse transcription PCR (qRT-PCR)

Total RNA was extracted from cells or grinded tumor lumps treated with trizol reagent (Invitrogen). Each sample was reverse transcribed into cDNA with the PrimeScript™RT Master Mix (TaKaRa). SYBR Green Realtime PCR Master Mix (TOYOBO, Osaka, Japan) and ABI 7500 real-time PCR system (Applied Biosystems) were used to measure the expression of target genes according to the recommendations of the manufacturer. Gene expression was calculated relative to β-actin, an internal reference gene, using the 2^−ΔΔct^ method. Primers were shown in Supplementary Table S3.

### Nuclear and cytoplasmic protein extraction

Extraction was performed using Nuclear and Cytoplasmic Protein Extraction Kit (Beyotime). Briefly, cells were resuspended in cytoplasmic protein isolation solution A with phenylmethanesulfonyl fluoride (PMSF) (Beyotime). Next, the homogenate was treated with cytoplasmic protein isolation solution B and centrifuged at 4 °C for 10 min. The obtained supernatant was cytoplasmic protein fraction. Then the precipitate was resuspended in nuclear protein isolation solution with PMSF, vortexed and homogenized on ice alternately for 30 min and centrifuged at 4 °C for 10 min. The supernatant was nuclear protein fraction.

### Western blotting assay

Protein was isolated from cells or grinded tumor lumps using RIPA Lysis Buffer (Beyotime) with PMSF. After measuring protein concentration by BCA Protrin Assay Kit (Beyotime), all the samples were boiled with 4 x SDS-PAGE Sample Loading Buffer (Beyotime) for 7 min at 100 °C. Then protein was separated by SDS-PAGE and transferred to PVDF membranes (Millipore, Darmstadt, Germany). Membranes were blocked by 5% milk and immunoblotted with primary antibodies (Supplementary information, Table S1). After incubation with HRP-labeled goat anti-mouse IgG or goat anti-rabbit IgG (Beyotime), the blots were detected using the Chemiluminescence Image Analysis System (Tanon, Shanghai, China) with ECL Iuminescence reagent (Sangon Biotech, Shanghai, China). β-actin and lamin B1 were used as loading control.

### Co-immunoprecipitation (co-IP) assay

Lysate of cells treated with SFE (20 µM) or overexpressing of GADD45B was generated under the addition of Halt Protease Inhibitor Cocktail (Thermo Fisher Scientific, Waltham, MA, USA) and Halt Phosphatase Inhibitor Cocktail (Thermo Fisher Scientific). Protein concentration was measured by the Pierce BCA Protein Assay Kit (Thermo Fisher Scientific). A total of 2 550 µg/mL protein was used for co-IP assay performed with the Pierce Co-IP Kit (Thermo Fisher Scientific). 50 µg of the GADD45B primary antibody was incubated with the delivered resin and covalently coupled for 2 h. The antibody-coupled resin was incubated with 250 µL cell lysates overnight at 4 °C, and then the protein complexes were eluted. Subsequent western blotting assay was performed as described before.

### Statistical analysis

Data were analyzed using the Prism software version 6 (GraphPad Software Inc., San Diego, CA, USA). Values are represented as the mean ± SD and all experiments were conducted three times. Significance was determined using the two-tailed Student’s *t*-test. *P* < 0.05 was considered statistically significant.

## Supplementary information

supplementary figure legends

Supplementary Information Figure S1

Supplementary Information Figure S2

Supplementary Information Figure S3

Supplementary Information Figure S4

Supplementary Information Figure S5

Supplementary Information Figure S6

Supplementary Information Figure S7

Supplementary Information Figure S8

Supplementary Information Figure S9

Supplementary Information Table S1

Supplementary Information Table S2

Supplementary Information Table S3
