# Supplementary material for: Sulforaphene inhibits esophageal cancer progression via suppressing SCD and CDH3 expression, and activating the GADD45B-MAP2K3-p38-p53 feedback loop
Source: Cell Death Dis. 2020 Sep 1;11(8):713. doi: 10.1038/s41419-020-02859-2 (PMC7463232; doi:10.1038/s41419-020-02859-2)
Supplement: Supplementary file 1 — supplementary figure legends [file 41419_2020_2859_MOESM1_ESM.doc]

**Supplementary Figure Legends:**

**Supplementary information, Figure S1 SFE inhibits EC109 and KYSE510 cell proliferation**. (A) Cell counting kit-8 reagent detection of the cell viability of EC109, KYSE510, KYSE150 and TE-1 cells with gradient concentrations of SFE (2.5 µM, 5 µM, 10 µM, 20 µM and 40 µM). IC50 was shown as mean ± s.d. (B) The activity of caspase-9, caspase-3 and caspase-8 in EC109 and KYSE510 cells was shown. (C) Representative images of mitochondrial membrane potential assays in EC109 and KYSE510 cells treated with SFE for different time or with gradient concentrations of SFE. CCCP, carbonyl cyanide 3-chlorophenylhydrazone, a positive control of decreased mitochondrial membrane potential. (D,E) Western blotting detection of protein involved in the mitochondrial apoptosis pathway (D) and G2/M phase (E) levels. Original magnification X100. Scale bars = 200 μm. Data represent the mean ± s.d. of three independent experiments. The statistical significance was assessed by Student's *t*-test. **P* < 0.05 and ***P* < 0.01. ns, not significant.

**Supplementary information, Figure S2 SCD and CDH3 have no effect on esophageal cancer cell proliferation**. (A,B) The effects of plasmids harboring full-length human SCD and CDH3 sequences named pC3.0-SCD (SCD) and pC3.0-CDH3 (CDH3), and siRNAs specific for SCD (si-SCD-1,si-SCD-2) and CDH3 (si-CDH3-1,si-CDH3-2) on SCD and CDH3 expression were measured by qRT-PCR and western blotting . (C,D) Cell counting (C) and colony formation assay (D) were carried out in EC109 and KYSE510 cells transfected with SCD and CDH3 plasmids, or siRNAs specific for SCD and CDH3. pC3.0, pcDNA3.0 vector plasmid. NC, negative control. Data represent the mean ± s.d. of three independent experiments. The statistical significance was assessed by Student's *t*-test. **P* < 0.05 and ***P* < 0.01. ns, not significant.

**Supplementary information, Figure S3 The effect of SCD and CDH3 on esophageal cancer cell metastasis**. (A) The scrape motility and transwell assays in EC109 and KYSE510 cells overexpressing SCD and CDH3. (B) The scrape motility and transwell assays in EC109 and KYSE510 cells decreasing SCD and CDH3 expression. Original magnification X100. Scale bars = 100 μm. Data represent the mean ± s.d. of three independent experiments. The statistical significance was assessed by Student's *t*-test. **P* < 0.05 and ***P* < 0.01.

**Supplementary information, Figure S4 SCD and CDH3 are positively related to the Wnt pathway.** (A) The gene set enrichment analysis (GSEA) analyses of SCD (upper) and CDH3 (lower) in esophageal tumor samples from LinkedOmics database were shown, ranked in order of NES from high to low. The colors indicate pathways positively correlated (blue) or negatively correlated (orange) with SCD and CDH3. The red lines indicate the Wnt pathway. (B) The GSEA plots from LinkedOmics database indicated the positive correlation between SCD (upper) or CDH3 (lower) and the Wnt pathway. NES, normalized enrichment score.

**Supplementary information, Figure S5 SCD and CDH3 can activate the Wnt** pathway**.** (A) qRT-PCR analyzed the mRNA levels of the Wnt pathway related genes in esophageal cancer cells. (B) Cytosolic and nuclear protein were isolated from SCD-, CDH3-slienced or SCD-, CDH3-overexpressing esophageal cancer cells, respectively, and used to analyze the Wnt pathway related gene expression by western blotting. Data represent the mean ± s.d. of three independent experiments. The statistical significance was assessed by Student's *t*-test. **P* < 0.05, ***P* < 0.01 and ****P* < 0.005. si-SCD, equal mixed si-SCD-1 and si-SCD-2. si-CDH3, equal mixed si-CDH3-1 and si-CDH3-2.

**Supplementary information, Figure S6 SFE can activate the p53 pathway through the p38 pathway.** (A) KEGG enrichment analysis of EC109 and KYSE510 microarray results was shown. The red lines indicate the p53 pathway. (B) qRT-PCR and western blotting indicated the mRNA, protein and phosphorylation levels of p53 in SFE-treated EC109 and KYSE510 cells. (C) Western blotting in EC109 and KYSE510 cells with SFE treatment. (D) The inhibitory effect of SB202190 on p38 and its downstream targets was measured by western blotting in EC109 and KYSE510 cells. (E)The effect of SFE treatment on the p38 pathway in EC109 and KYSE510 cells which had been treated with 30 µM SB202190 for 6 h was measured by western blotting. Data represent the mean ± s.d. of three independent experiments. The statistical significance was assessed by Student's *t*-test. *P < 0.05, ***P* < 0.01 and ****P* < 0.005. ns, not significant.

**Supplementary information, Figure S7 MAP2K3 and GADD45B can inhibit the proliferation of esophageal cancer cells.** (A,B) qRT-PCR and western blotting were applied to measure MAP2K3 and GADD45B expression in EC109 and KYSE510 cells. (C,D) The effect of MAP2K3 and GADD45B overexpressing or down-regulated on EC109 and KYSE510 cell proliferation was measured by cell counting (C) and colony formation assay (D). Data represent the mean ± s.d. of three independent experiments. The statistical significance was assessed by Student's *t*-test. **P* < 0.05, ***P* < 0.01 and ****P* < 0.005.

**Supplementary information, Figure S8 Silencing MAP2K3 and GADD45B can inhibit the effect of SFE on esophageal cancer cell proliferation.** (A,B) Flow cytometry analysis detected cell apoptosis (A) and cell cycle (B) in EC109 and KYSE510 cells. (C,D) Western blotting was applied to detected protein related to apoptosis and G2/M phase expression in EC109 and KYSE510 cells. Data represent the mean ± s.d. of three independent experiments. si-MAP2K3, equal mixed si-MAP2K3-1 and si-MAP2K3-2. si-GADD45B, equal mixed si-GADD45B-1 and si-GADD45B-2. The statistical significance was assessed by Student's *t*-test. **P* < 0.05 and ***P* < 0.01.

**Supplementary information, Figure S9 The relationship among GADD45B, MAP2K3, p38 and p53.** (left) The STRING database demonstrated the protein-protein interaction network involving GADD45B, MAP3K4 and MAP2K3. (right) A positive feedback loop consisted of GADD45B, MAP2K3, p38 and p53 in esophageal cancer cells.
