# Supplementary figures and images for: Sulforaphene inhibits esophageal cancer progression via suppressing SCD and CDH3 expression, and activating the GADD45B-MAP2K3-p38-p53 feedback loop
Source: Cell Death Dis. 2020 Sep 1;11(8):713. doi: 10.1038/s41419-020-02859-2 (PMC7463232; doi:10.1038/s41419-020-02859-2)

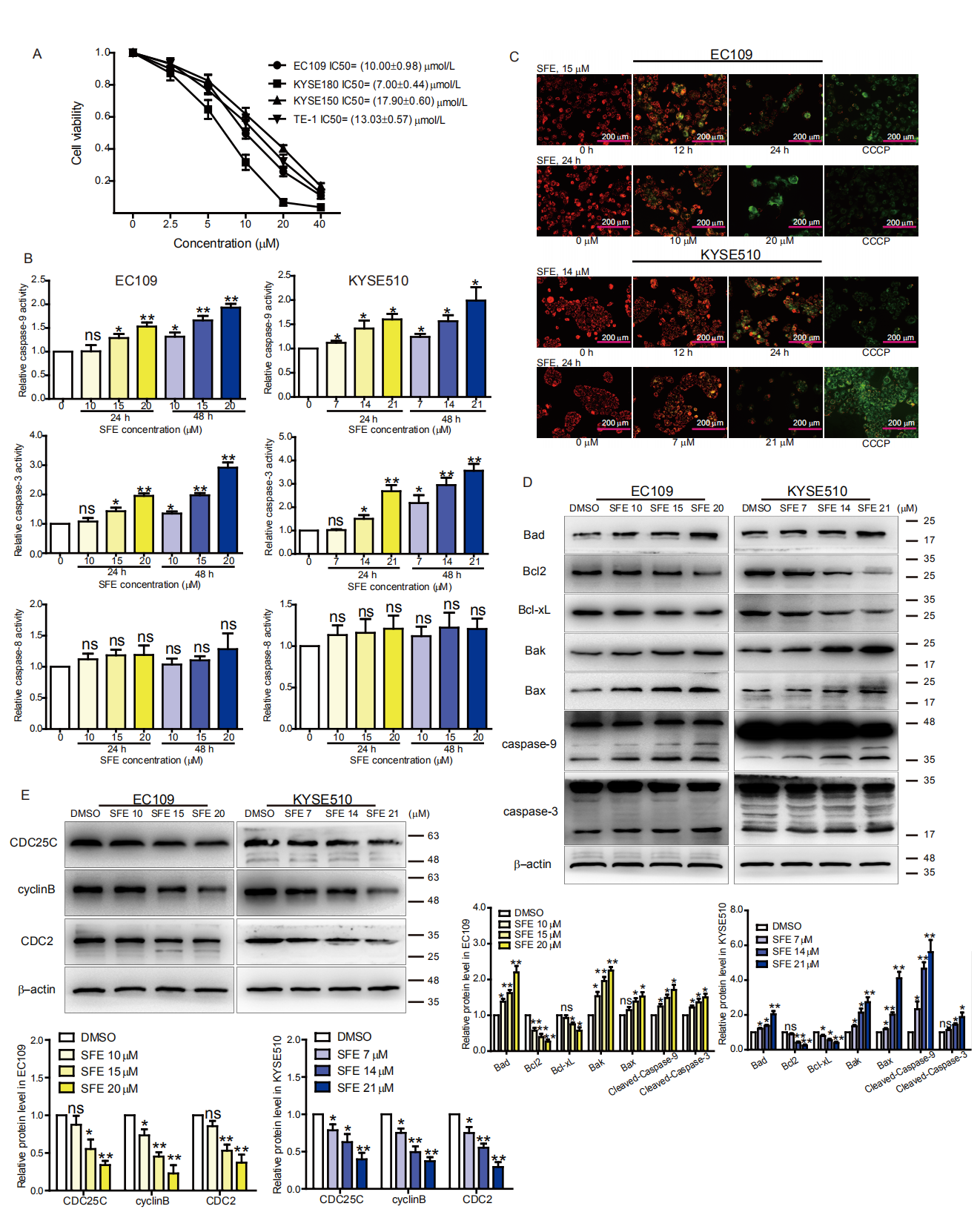

Supplement: Supplementary file 2 — Supplementary Information Figure S1 [file 41419_2020_2859_MOESM2_ESM.png]

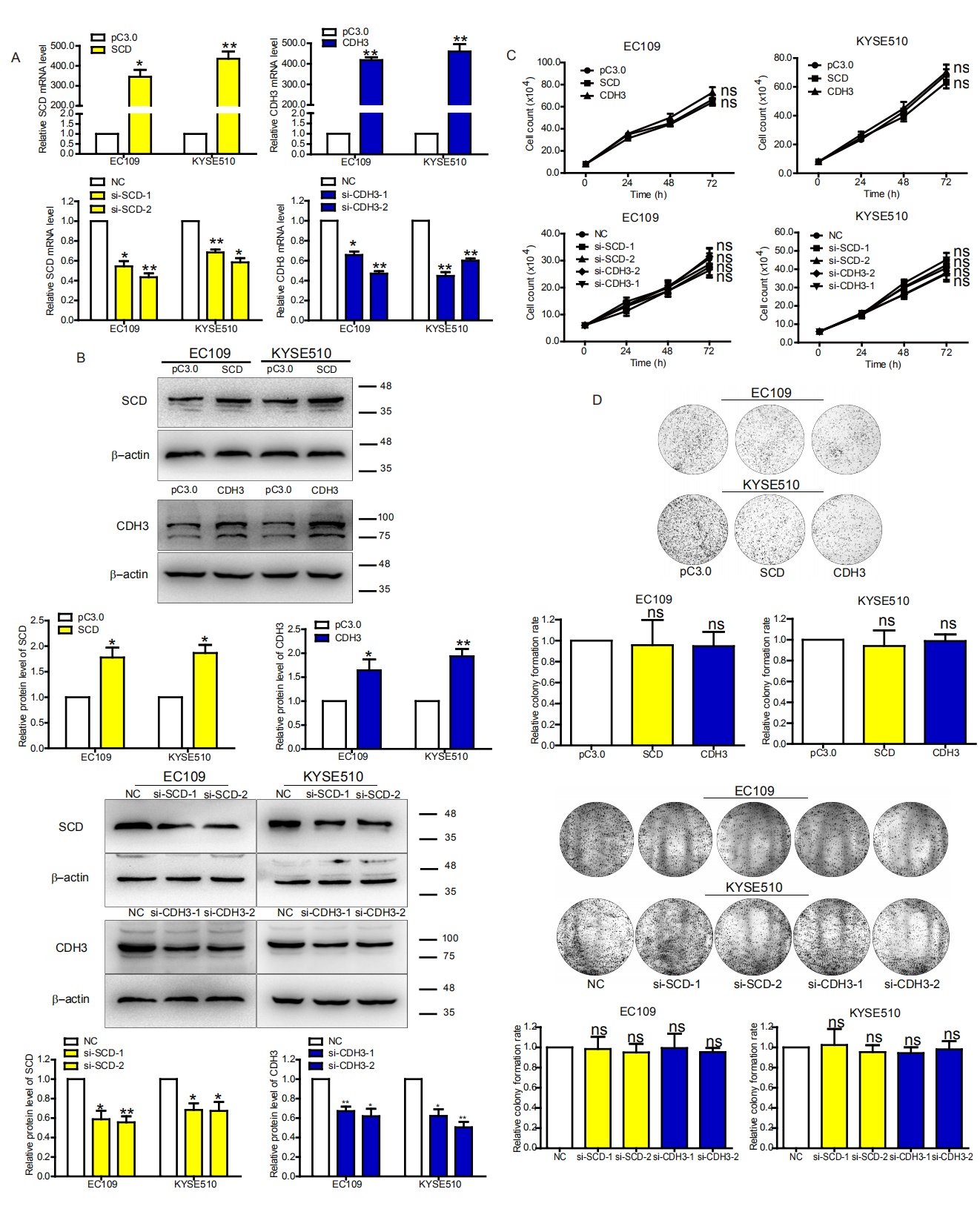

Supplement: Supplementary file 3 — Supplementary Information Figure S2 [file 41419_2020_2859_MOESM3_ESM.png]

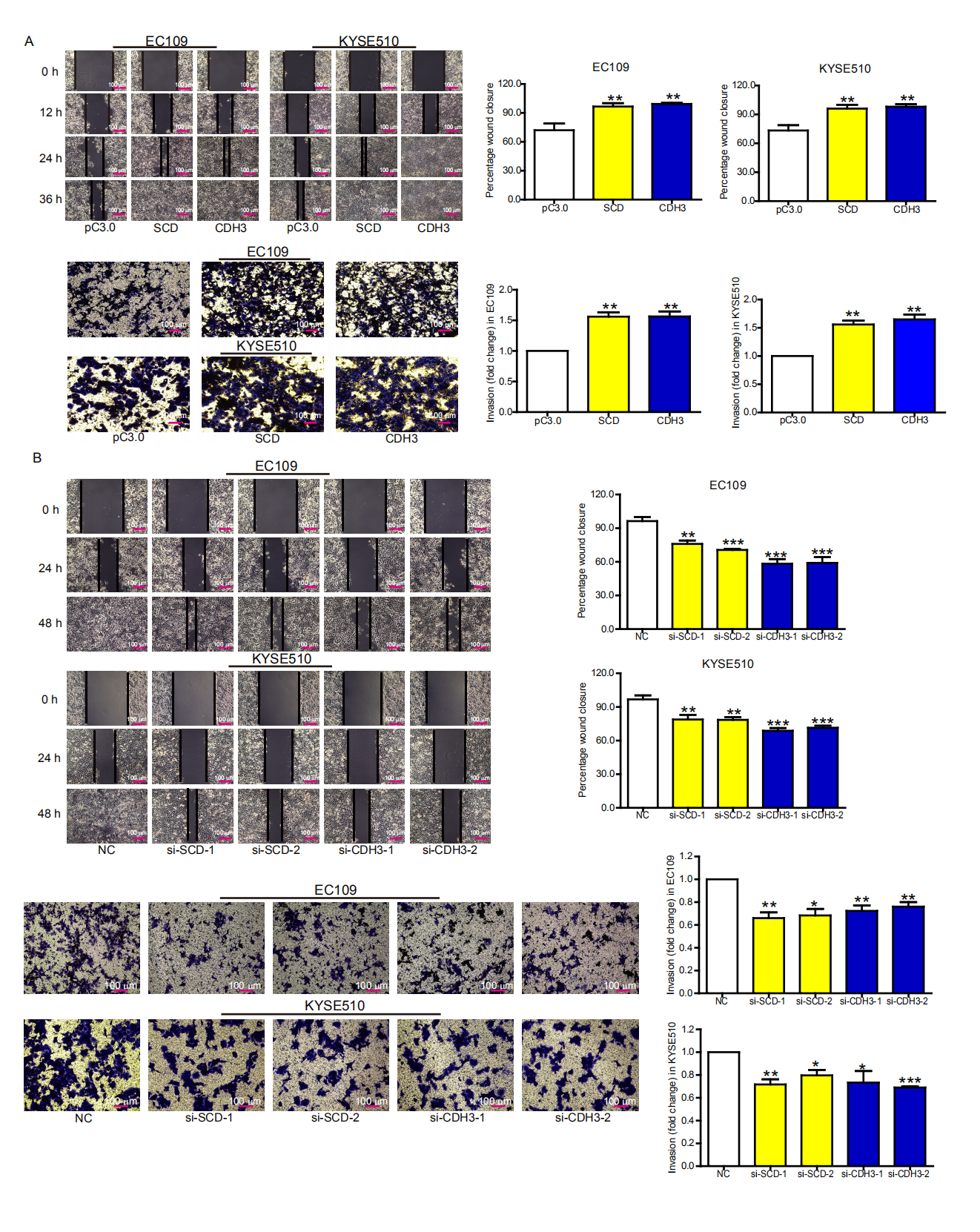

Supplement: Supplementary file 4 — Supplementary Information Figure S3 [file 41419_2020_2859_MOESM4_ESM.png]

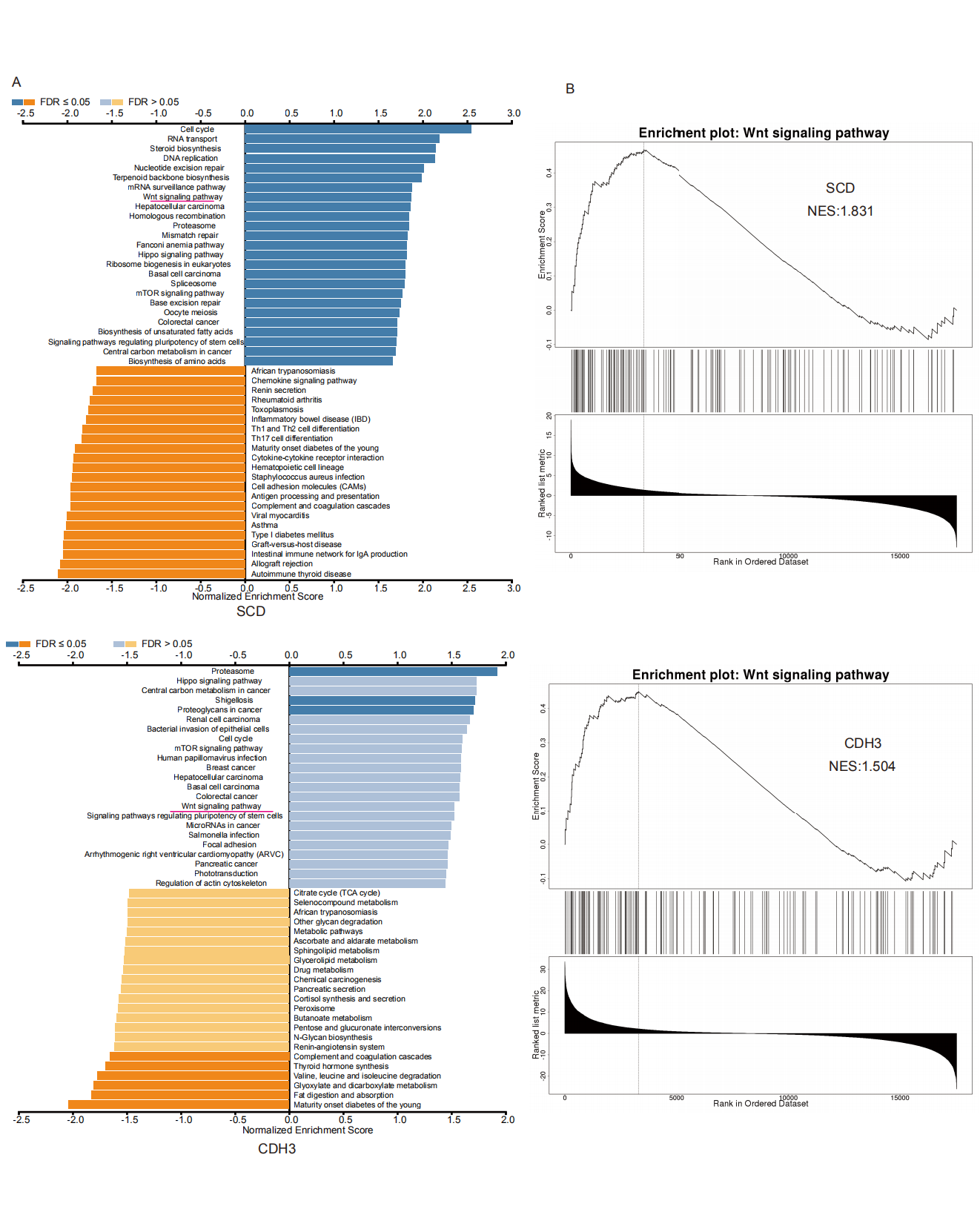

Supplement: Supplementary file 5 — Supplementary Information Figure S4 [file 41419_2020_2859_MOESM5_ESM.png]

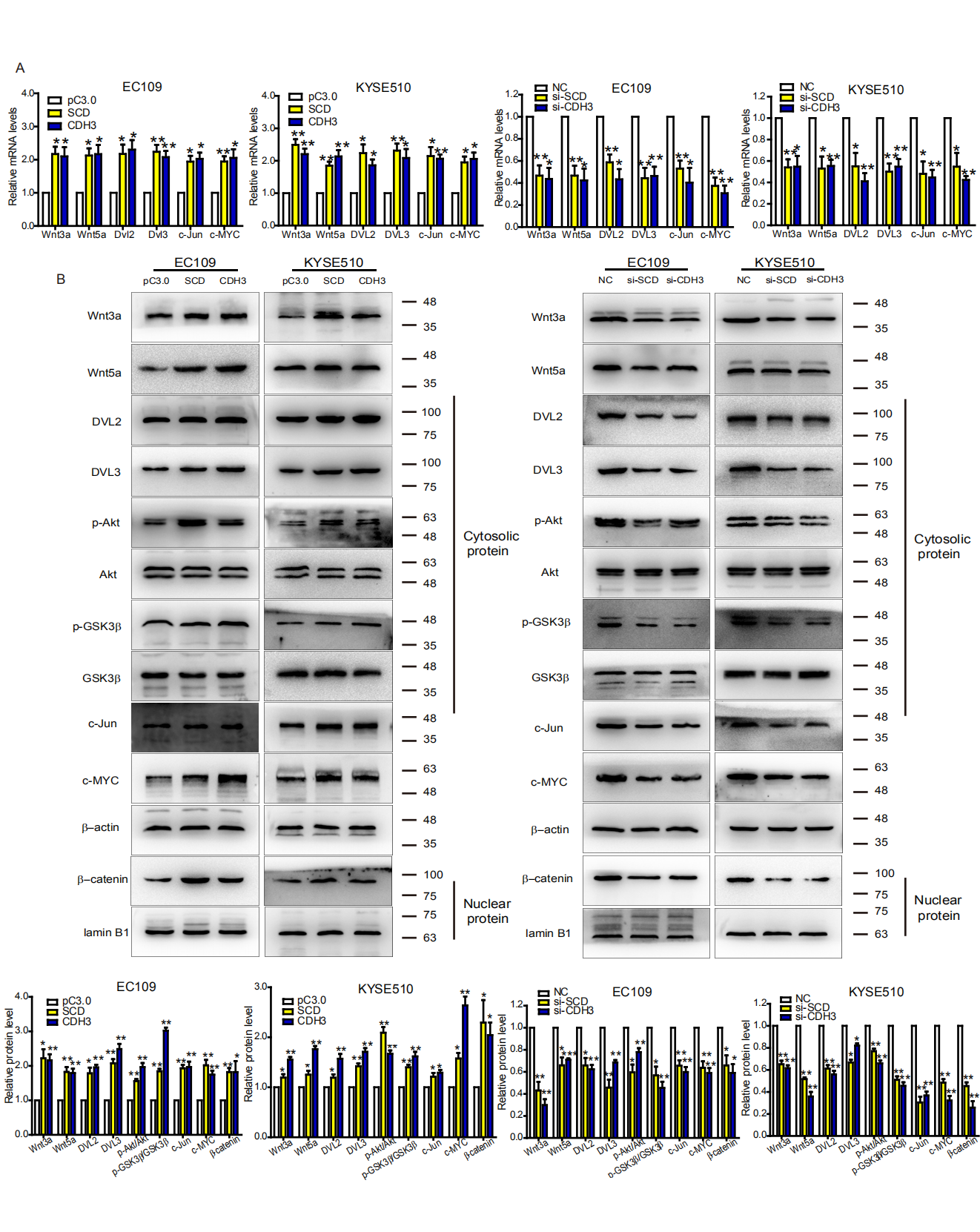

Supplement: Supplementary file 6 — Supplementary Information Figure S5 [file 41419_2020_2859_MOESM6_ESM.png]

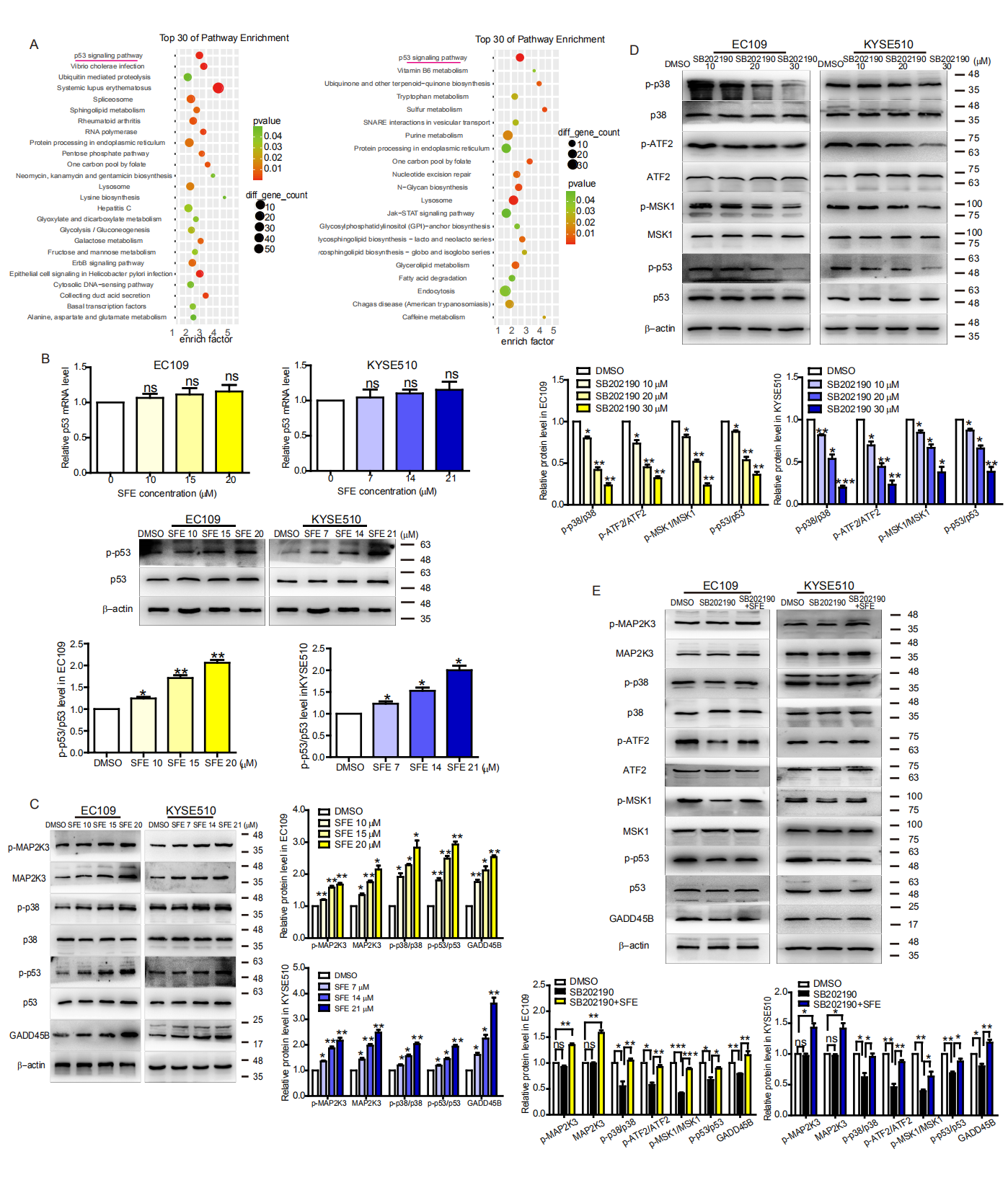

Supplement: Supplementary file 7 — Supplementary Information Figure S6 [file 41419_2020_2859_MOESM7_ESM.png]

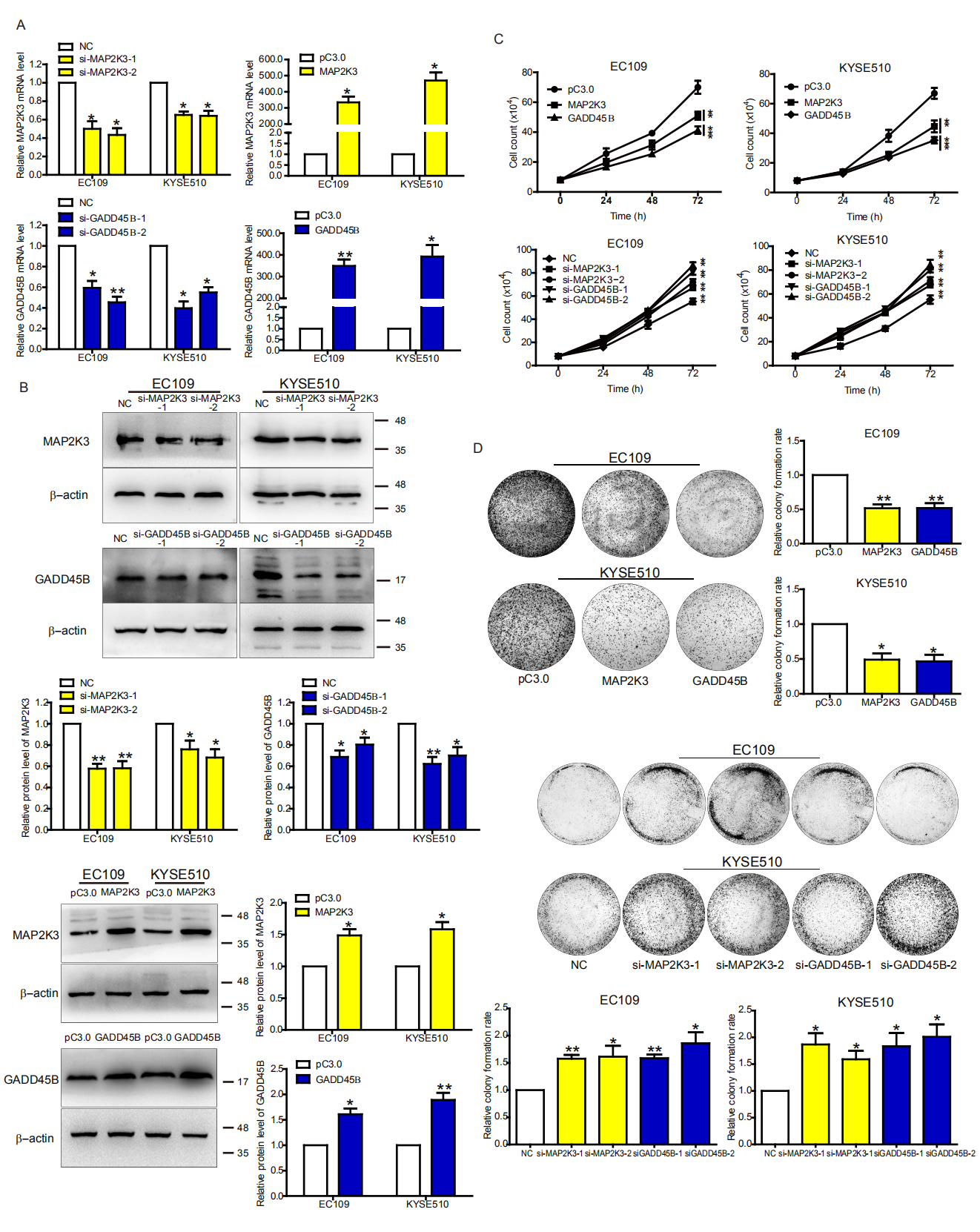

Supplement: Supplementary file 8 — Supplementary Information Figure S7 [file 41419_2020_2859_MOESM8_ESM.png]

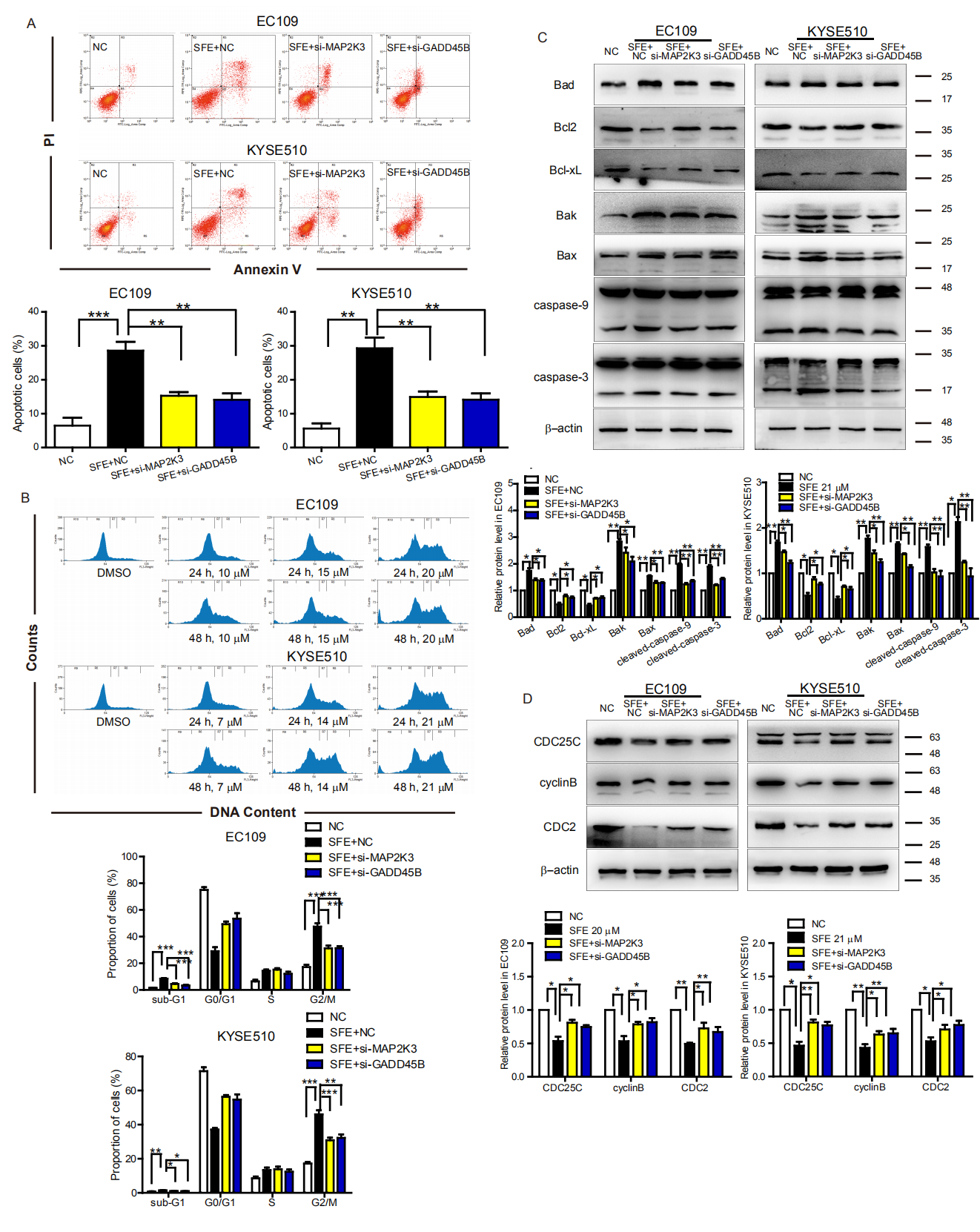

Supplement: Supplementary file 9 — Supplementary Information Figure S8 [file 41419_2020_2859_MOESM9_ESM.png]

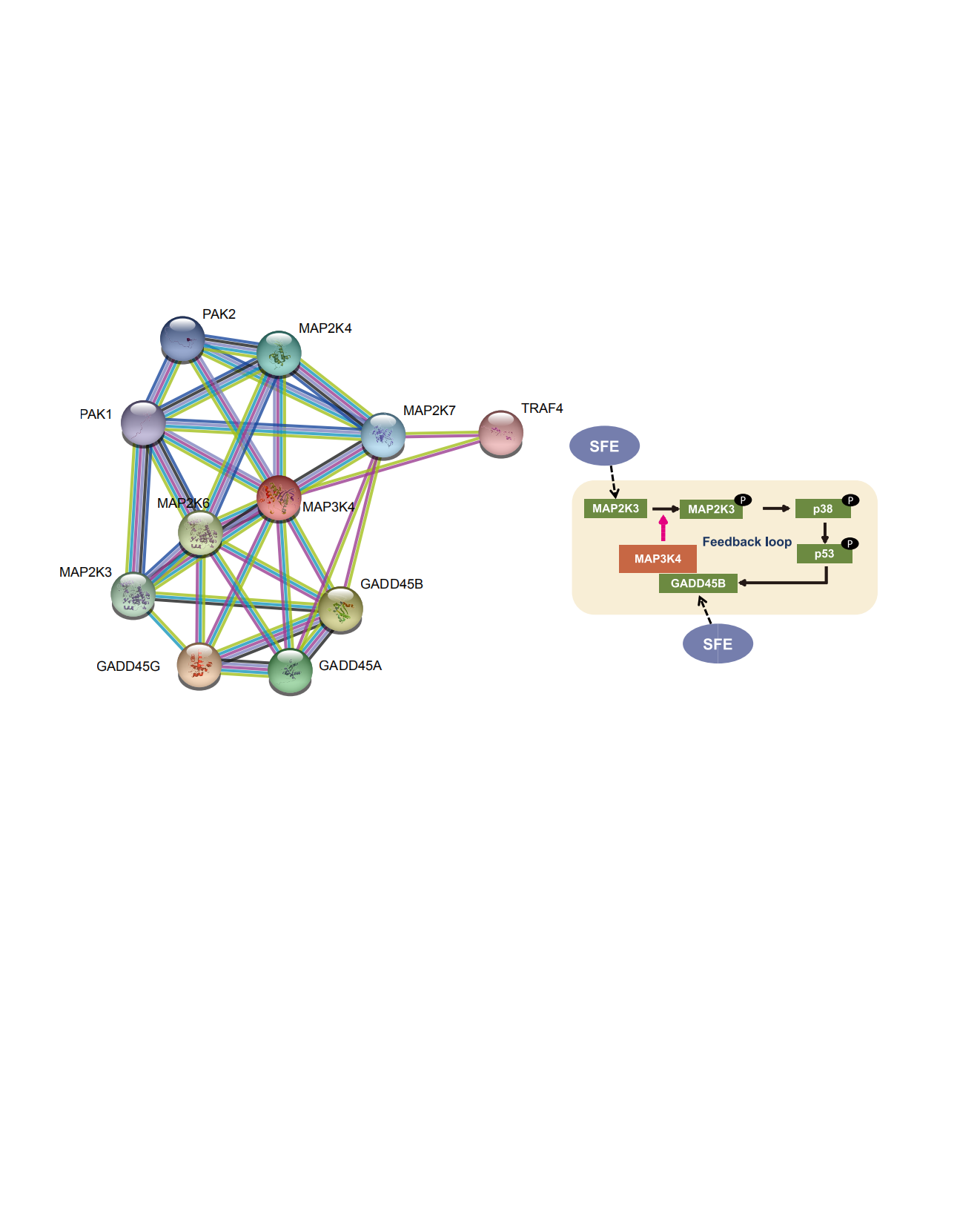

Supplement: Supplementary file 10 — Supplementary Information Figure S9 [file 41419_2020_2859_MOESM10_ESM.png]
