# Supplementary material for: Sulforaphene inhibits esophageal cancer progression via suppressing SCD and CDH3 expression, and activating the GADD45B-MAP2K3-p38-p53 feedback loop
Source: Cell Death Dis. 2020 Sep 1;11(8):713. doi: 10.1038/s41419-020-02859-2 (PMC7463232; doi:10.1038/s41419-020-02859-2)
Supplement: Supplementary file 11 — Supplementary Information Table S1 [file 41419_2020_2859_MOESM11_ESM.doc]

**Supplementary information, Table S1. Primary antibodies used in this study.**

| Name | Catalog number | Manufacture | |
| --- | --- | --- | --- |
| Bad | 9292 | Cell Signaling Technology | Danvers, MA, USA |
| Bcl2 | 12789-1-AP | Proteintech | Wuhan, China |
| Bcl-xL | 10783-1-AP | Proteintech | Wuhan, China |
| Bak | 12105 | Cell Signaling Technology | Danvers, MA, USA |
| Bax | 5023 | Cell Signaling Technology | Danvers, MA, USA |
| caspase-9 | 10380-1-AP | Proteintech | Wuhan, China |
| caspase-3 | 19677-1-AP | Proteintech | Wuhan, China |
| CDC25C | 16485-1-AP | Proteintech | Wuhan, China |
| cyclinB | 12231 | Cell Signaling Technology | Danvers, MA, USA |
| CDC2 | 19532-1-AP | Proteintech | Wuhan, China |
| N-cadherin | 13116 | Cell Signaling Technology | Danvers, MA, USA |
| vimentin | 5741 | Cell Signaling Technology | Danvers, MA, USA |
| Snail1 | 3879 | Cell Signaling Technology | Danvers, MA, USA |
| Slug | 9585 | Cell Signaling Technology | Danvers, MA, USA |
| E-cadherin | 3195 | Cell Signaling Technology | Danvers, MA, USA |
| MUC1 | 14161 | Cell Signaling Technology | Danvers, MA, USA |
| SCD | 2794 | Cell Signaling Technology | Danvers, MA, USA |
| CDH3 | 13773-1-AP | Proteintech | Wuhan, China |
| Wnt3a | 2721 | Cell Signaling Technology | Danvers, MA, USA |
| Wnt5a | ab153876 | Abcam | Cambridge, MA, USA |
| DVL2 | 3224 | Cell Signaling Technology | Danvers, MA, USA |
| DVL3 | 13444-1-AP | Proteintech | Wuhan, China |
| p-Akt (Ser473) | 4060 | Cell Signaling Technology | Danvers, MA, USA |
| Akt | 60203-2-lg | Proteintech | Wuhan, China |
| p-GSK3β (Ser9) | 5558 | Cell Signaling Technology | Danvers, MA, USA |
| GSK3β | 22104-1-AP | Proteintech | Wuhan, China |
| c-Jun | 9165 | Cell Signaling Technology | Danvers, MA, USA |
| c-MYC | 5605 | Cell Signaling Technology | Danvers, MA, USA |
| β-catenin | 8480 | Cell Signaling Technology | Danvers, MA, USA |
| MAP2K3 | ab195037 | Abcam | Wuhan, China |
| GADD45B | ab105060 | Abcam | Cambridge, MA, USA |
| p-MAP2K3 (Ser189) | ab194809 | Abcam | Cambridge, MA, USA |
| p-p38 (Thr180/Tyr182) | 4511 | Cell Signaling Technology | Danvers, MA, USA |
| p38 | 14064-1-AP | Proteintech | Wuhan, China |
| p-p53 (Ser33) | 2526 | Cell Signaling Technology | Danvers, MA, USA |
| p53 | 10442-1-AP | Proteintech | Wuhan, China |
| MAP3K4 | ab186125 | Abcam | Cambridge, MA, USA |
| p-ATF2 (Thr71) | 24329 | Cell Signaling Technology | Danvers, MA, USA |
| ATF2 | 35031 | Cell Signaling Technology | Danvers, MA, USA |
| p-MSK1 (Thr581) | 9595 | Cell Signaling Technology | Danvers, MA, USA |
| MSK1 | ab155405 | Abcam | Cambridge, MA, USA |
| lamin B1 | 12987-1-AP | Proteintech | Wuhan, China |
| β-actin | 66009-1-Ig | Proteintech | Wuhan, China |
