# Supplementary material for: Sulforaphene inhibits esophageal cancer progression via suppressing SCD and CDH3 expression, and activating the GADD45B-MAP2K3-p38-p53 feedback loop
Source: Cell Death Dis. 2020 Sep 1;11(8):713. doi: 10.1038/s41419-020-02859-2 (PMC7463232; doi:10.1038/s41419-020-02859-2)
Supplement: Supplementary file 12 — Supplementary Information Table S2 [file 41419_2020_2859_MOESM12_ESM.doc]

**Supplementary information, Table S2. siRNAs, negative control, miR-29a-5p mimics and miR-29a-5p inhibitor sequences used in this study**

| Name |  | siRNA sequence (5' to 3') |
| --- | --- | --- |
| SCD siRNA-1 | Sense | CUACGGCUCUUUCUGAUCATT |
|  | Antisense | UGAUCAGAAAGAGCCGUAGTT |
| SCD siRNA-2 | Sense | GGUUGAAUAUGUCUGGAGATT |
|  | Antisense | UCUCCAGACAUAUUCAACCTT |
| CDH3 siRNA-1 | Sense | GGAAUCCAUUGAAGAUCUUTT |
|  | Antisense | AAGAUCUUCAAUGGAUUCCTT |
| CDH3 siRNA-2 | Sense | CCGUGAGGAUGAGCAGUUUTT |
|  | Antisense | AAACUGCUCAUCCUCACGGTT |
| MAP2K3 siRNA-1 | Sense | GCUACAAUGUCAAGUCCGATT |
|  | Antisense | UCGGACUUGACAUUGUAGCTT |
| MAP2K3 siRNA-2 | Sense | GAAGAAGGAUCUACGGAUATT |
|  | Antisense | UAUCCGUAGAUCCUUCUUCTT |
| GADD45B siRNA-1 | Sense | GAACUUGGUUGGUCCUUGUTT |
|  | Antisense | ACAAGGACCAACCAAGUUCTT |
| GADD45B siRNA-2 | Sense | GUGACAACGACAUCAACAUTT |
|  | Antisense | AUGUUGAUGUCGUUGUCACTT |
| Negative control | Sense | UUCUCCGAACGUGUCACGUTT |
|  | Antisense | ACGUGACACGUUCGGAGAATT |
| miR-29a-5p mimics |  | ACUGAUUUCUUUUGGUGUUCAG |
| miR-29a-5p inhibitor |  | CUGAACACCAAAAGAAAUCAGU |
