# Supplementary material for: Sulforaphene inhibits esophageal cancer progression via suppressing SCD and CDH3 expression, and activating the GADD45B-MAP2K3-p38-p53 feedback loop
Source: Cell Death Dis. 2020 Sep 1;11(8):713. doi: 10.1038/s41419-020-02859-2 (PMC7463232; doi:10.1038/s41419-020-02859-2)
Supplement: Supplementary file 13 — Supplementary Information Table S3 [file 41419_2020_2859_MOESM13_ESM.doc]

**Supplementary information, Table S3. Primer sequence for qRT-PCR**

| Primers | Sequence (5' to 3') |
| --- | --- |
| IFNL2-F | CCAAGCGTCACCATGCTTTC |
| IFNL2-R | CCAGACCTCAGTCCCTCTCTT |
| OASL-F | CTGATGCAGGAACTGTATAGCAC |
| OASL-R | CACAGCGTCTAGCACCTCTT |
| IFNL1-F | CACATTGGCAGGTTCAAATCTCT |
| IFNL1-R | CCAGCGGACTCCTTTTTGG |
| IFNL3-F | TAAGAGGGCCAAAGATGCCTT |
| IFNL3-R | CTGGTCCAAGACATCCCCC |
| CCL5-F | CCAGCAGTCGTCTTTGTCAC |
| CCL5-R | CTCTGGGTTGGCACACACTT |
| IL1A-F | TGGTAGTAGCAACCAACGGGA |
| IL1A-R | ACTTTGATTGAGGGCGTCATTC |
| HIST2H2AB-F | CCATCTGCAACTAGCCGTGAG |
| HIST2H2AB-R | CAGGCTTGTGACTCTCCGT |
| SCD-F | TCTAGCTCCTATACCACCACCA |
| SCD-R | TCGTCTCCAACTTATCTCCTCC |
| KRT6A-F | CCTGGCACTGAGCACAACTT |
| KRT6A-R | GTGAGGGCACTAAGCATCCA |
| CAPNS2-F | AGTGAGGAAGTTAGGCGATTTCG |
| CAPNS2-R | GGTGTCAAGACTAAAACCGTCA |
| CXCL8-F | ACTGAGAGTGATTGAGAGTGGAC |
| CXCL8-R | AACCCTCTGCACCCAGTTTTC |
| HIST1H2BM-F | GAAAGAAGCGCAAACGCAG |
| HIST1H2BM-R | TGATTCCCATAGCCTTGGAAGA |
| CDH3-F | TGGAGATCCTTGATGCCAATGA |
| CDH3-R | GCGTCCAGATCAGTGACCG |
| KRT6B-F | GGAGTGCAGGTGGGTAACTG |
| KRT6B-R | CCTCAGTGGGTGGGAAAGTC |
| HIST1H2BF-F | ACCTGCTAAGTCCGCTCCT |
| HIST1H2BF-R | CTACGCTTGCGCTTCTTACCA |
| TNFRSF10D-F | TACCACGACCAGAGACACC |
| TNFRSF10D-R | CACCCTGTTCTACACGTCCG |
| HSPH1-F | ACAGCCATGTTGTTGACTAAGC |
| HSPH1-R | GCATCTAACACAGATCGCCTCT |
| HSPB8-F | CTCCTGCCACTACCCAAGC |
| HSPB8-R | GGCCAAGAGGCTGTCAAGT |
| KRTAP2-3-F | CTCTCCTCTCAACGCACGAA |
| KRTAP2-3-R | TGTCAGAGAGGGCCAGGATT |
| DEFB103A-F | TCCTTCAGAGTGCTTTGGACC |
| DEFB103A-R | CTCAGAGGTAGCCCCAACAC |
| ANKRD1-F | AGTAGAGGAACTGGTCACTGG |
| ANKRD1-R | TGTTTCTCGCTTTTCCACTGTT |
| SMCR8-F | CCTGACGTAGTGGCCTTCAC |
| SMCR8-R | CCCCGGACAGTTTTGACCA |
| HIF1A-AS2-F | GACCTAAGGCTCTGGCACTT |
| HIF1A-AS2-R | AACATCTTCTGTGGACCAGGC |
| TIMM17A-F | GGTGGGGCCTTTACGATGG |
| TIMM17A-R | GCCCTGGTTTTAATAGCTGTCA |
| ARL6IP1-F | TGCTGATGGCTGATAAAGTCCT |
| ARL6IP1-R | AAACGCCGGACAGAACAGATG |
| ATP13A3-F | GGGACAACTGTTATTCAGACTCG |
| ATP13A3-R | GCAACTGCCACAAGACATAGTA |
| SNORD11B-F | TCTGATGGCAATGATGATTTTTACA |
| SNORD11B-R | TGAGGTAGTGACTGAACACCC |
| CYR61-F | ACCGCTCTGAAGGGGATCT |
| CYR61-R | ACTGATGTTTACAGTTGGGCTG |
| DDX3X-F | AGCAGTTTTGGATCTCGTAGTG |
| DDX3X-R | ACTGTTTCCACCACGTTCAAAT |
| MAP2K3-F | GAGGGAGACGTGTGGATCTG |
| MAP2K3-R | GTGAGGGCACTAAGCATCCA |
| GADD45B-F | TACGAGTCGGCCAAGTTGATG |
| GADD45B-R | GGATGAGCGTGAAGTGGATTT |
| p53-F | CAGCACATGACGGAGGTTGT |
| p53-R | TCATCCAAATACTCCACACGC |
| β-actin-F | GGCGGCACCACCATGTACCCT |
| β-actin-R | AGGGGCCGGACTCGTCATACT |
